# Perinatal bereavement rooms: a narrative review of physical space in perinatal grief

**DOI:** 10.1007/s00404-025-08175-4

**Published:** 2025-09-23

**Authors:** Ruby Castilla-Puentes, Azul F. Isidoro, Alfonsina Orosito, Samantha Eaton, Manuela Goyeneche, Liliana González Cabrales, Gabriela Santaella

**Affiliations:** 1 University of Cincinnati, WARMI Mental Health, 7709 Cornell Rd, Cincinnati, OH 45242 USA; 2 University of Cincinnati, 2600 Clifton Ave, Cincinnati, OH 45221 USA; 3 Aalborg University, Fredrik Bajers Vej 7K, 9220 Aalborg Øst, Denmark; 4 National University of Cuyo (UNCuyo), M5502JMA Mendoza, Argentina; 5 University of Washington, 1410 NE Campus Pkwy, Seattle, WA 98195 USA; 6 Pontificia Universidad Javeriana, Kra 7 #40-62, Bogotá, Colombia; 7 Carolinas Medical Center- Atrium Health, 501 Billingsley Rd, Charlotte, NC 28211 USA

**Keywords:** Perinatal grief rooms, Perinatal death, Bereavement rooms

## Abstract

**Background:**

Perinatal loss is a profoundly complex form of grief, often linked to heightened risk of prolonged bereavement and adverse mental health outcomes. Perinatal grief rooms—private, supportive spaces within healthcare settings—aim to help families process their loss, spend time with their baby, and create meaningful memories in a respectful environment. While bereavement care has received growing attention, the role of the physical environment in supporting grief remains underexplored.

**Objective:**

To synthesize current evidence on how dedicated physical spaces can support individuals and families after perinatal loss, and to identify priorities for research, design standards, and interdisciplinary collaboration.

**Methods:**

A narrative review was conducted in accordance with PRISMA-ScR guidelines. Literature searches were performed across PubMed, PsycINFO, Medline (OVID), Embase, ScienceDirect, SCOPUS, SciELO, and Google Scholar using terms, such as “perinatal grief rooms”, “bereavement rooms”, “angel suites”, “butterfly suites”, “snowdrop suites”, “cloud rooms”, “designated units for perinatal loss”, and “birthing + bereavement suites”. The review examined (1) the current role of physical spaces in the perinatal loss experience, and (2) how their availability and design may influence grief outcomes.

**Results:**

Of the 17 articles meeting inclusion criteria, only 4 (24%) referenced bereavement rooms, and just 3 (18%) noted the need for formal protocols—without offering concrete examples. No studies evaluated implementation, design standards, or measurable impact on grief, mental health, or family well-being. This lack of empirical evidence and standardized guidance underscores a critical gap that limits integration of therapeutic environments into perinatal bereavement care.

**Conclusion:**

Despite increasing recognition of the importance of bereavement care, dedicated grief rooms remain under-researched and inconsistently implemented. Advancing this field will require rigorously designed studies, development of design standards, and collaborative partnerships among healthcare providers, researchers, policymakers, and design experts to ensure equitable access to therapeutic spaces for grieving families.

## Introduction

Perinatal loss—encompassing miscarriage, stillbirth, and neonatal death—has profound and lasting impacts on parents, families, and healthcare providers. Extensive research indicates that grief following the loss of a child during the perinatal period is among the most complex and profound challenges an individual may face [6]. Although research on bereavement care after perinatal loss has expanded in recent years, there remains a significant gap in understanding how the physical environment—such as dedicated rooms, suites, or other infrastructure—affects the grieving process following miscarriage, stillbirth, or neonatal death.

The distinctive nature of perinatal grief, compared with other forms of bereavement, can lead to more intense or complicated grieving processes [22]. Women who experience loss during pregnancy are at increased risk of mental health conditions, such as depression, anxiety, posttraumatic stress disorder, and/or complicated grief [2, 12, 30]. According to research by Obst and colleagues, complicated grief scores within the clinical range were higher among perinatally bereaved individuals than in many other bereaved populations [19]. Clinical practice guidelines identify providing a separate room, ensuring privacy, and allowing time with the baby as essential components of bereavement care following perinatal loss (John [13, 24]).

Perinatal grief rooms provide a quiet, private environment where parents can mourn and spend time with their baby. These spaces are designed to accommodate the needs of the entire family, including siblings and other relatives, and to support the creation of lasting memories—such as hand and foot castings, photographs, or written letters [23]. In this study, we review published literature on these spaces, examine how they have influenced prenatal clinical care, and consider future directions.

For the purposes of this study, the term *bereavement room* refers to a designated area within a hospital or healthcare facility intended for families who have experienced the death of a fetus or newborn. Other terms identified in the literature include *perinatal grief rooms*, *bereavement suites*, *angel suites*, *butterfly suites*, *snowdrop suites*, *cloud rooms*, and *designated units for perinatal loss*. In our search strategy, we also included the term “*birthing* + *bereavement suites*” to capture variations in phrasing. These terms are used interchangeably to describe the same type of space.

Despite increasing recognition of the importance of supportive environments in perinatal bereavement care, there is a critical lack of empirical research, standardized guidelines, and implementation strategies for dedicated bereavement rooms within healthcare settings. This review seeks to map the current state of knowledge, identify existing gaps, and provide a foundation for future evidence-based recommendations.

## Materials and methods

### Narrative review process

This study was conducted as a narrative review to provide a comprehensive and critical overview of the topic. The narrative review approach offers flexibility for a nuanced examination and critique of current literature, making it well suited to present a broad perspective. This is particularly appropriate for exploring the role of physical space in perinatal grief, where the available evidence is limited, varied in scope, and dispersed across multiple disciplines.

To ensure methodological rigor and transparency, the review followed the framework outlined by Levac et al., which builds on Arksey & O’Malley’s scoping review methodology and consists of the following five stages [16]:

1. Identifying the research question
2. Identifying relevant studies
3. Selecting studies
4. Charting the data
5. Collating, summarizing, and reporting the results.

Each stage is described in the sections below.

### Stage 1: identifying the research question

The review was guided by the following research questions:

1. What is currently known about the role of physical spaces in the perinatal loss experience?
2. How does the availability of such spaces influence grief among individuals and families who have experienced miscarriage, stillbirth, or neonatal death?

These questions were designed to be broad enough to capture diverse literature but focused enough to inform practice and highlight knowledge gaps. They shaped the search strategy, study selection, and synthesis processes.

### Stage 2: identifying relevant studies

A comprehensive literature search was performed following the Preferred Reporting Items for Systematic Reviews and Meta-Analyses extension for Narrative Reviews (PRISMA-ScR) guidelines. The following databases were searched: PubMed, PsycINFO, Medline (OVID), Embase, ScienceDirect, SCOPUS, SciELO, and Google Scholar.

Search terms included combinations of: “*perinatal grief rooms”*, *“bereavement rooms”*, *“bereavement suites”*, *“angel suites”*, *“butterfly suites”*, *“snowdrop suites”*, *“cloud rooms”*, *“designated units for perinatal loss”, and “birthing* + *bereavement suites”* (Table 1).

**Table 1:** List of terms used in the search strategy by language (English and Spanish)

English	Spanish
Perinatal grief rooms	Salas de duelo perinatal
Bereavement rooms	Habitaciones de duelo
Bereavement suites	Suites de duelo
Angel suites	Suites de ángeles
Butterfly suites	Suites de mariposas
Snowdrop suites	Suites de Copos de Nieve
Cloud rooms	Habitaciones de nubes
Designated units for perinatal loss	Unidades designadas para pérdida perinatal
Birthing + bereavement suites	Suites de parto y duelo

Boolean operators were applied as follows: (Perinatal OR neonatal OR fetal) AND (loss OR stillbirth OR fetal death OR neonatal death) AND (room OR suites OR grief room OR bereavement suite OR perinatal grief rooms).

Inclusion criteria:

- Peer-reviewed articles published in English or Spanish
- Studies describing physical spaces for perinatal grief within healthcare facilities
- Research including parental perspectives on the need for dedicated spaces
- Studies exploring parental mental health outcomes associated with these environments
- Websites of relevant non-profit organizations and other entities promoting or supporting such spaces.

Exclusion criteria:

- Sources describing spaces of remembrance or grief outside healthcare facilities (e.g., cemeteries, funeral homes, or other non-clinical settings)

Rationale for including websites and organizations: Due to the limited number of peer-reviewed studies directly addressing perinatal grief rooms, reputable websites and organizational reports were included to capture current initiatives, design guidelines, and emerging practices that may not yet be represented in the academic literature. Selection of the 5 websites and 3 organizations was based on:

1. Relevance to perinatal bereavement care,
2. Provision of specific information on physical room design or use, and
3. Accessibility of source content for full review.

For this review, *perinatal loss* was defined as miscarriage, stillbirth, neonatal death, fetal death, or infant death.

### Stage 3: selecting studies

**Fig. 1:**
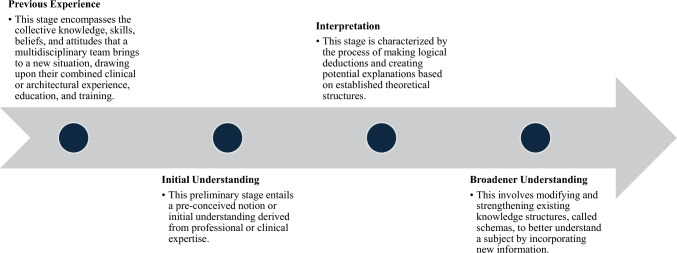
Steps of the hermeneutic process—narrative review of physical space in perinatal grief

All sources meeting the eligibility criteria were reviewed in full using a hermeneutic process (Fig. 1). This interpretive method analyzes and understands texts within their historical, cultural, and contextual backgrounds, moving iteratively between the parts and the whole to uncover nuanced meaning [10]. It is particularly valuable for topics with limited prior research, where context is essential to interpretation.

The review process involved two independent reviewers (RC and AO) evaluating each source in parallel. Discrepancies were discussed and, if unresolved, adjudicated by a third reviewer (MG). This process recognized that meaning is constructed rather than fixed and sought to incorporate diverse disciplinary perspectives relevant to perinatal grief and the role of physical space.

### Stage 4: charting the data

References to perinatal grief rooms identified within the articles were systematically extracted. Key study details—including publication year, study type, country, setting, and main findings—were organized and are summarized in Table 2.

**Table 2:** Summary of scientific studies about perinatal loss and bereavement rooms

First author/reference	Title (English/Spanish)	Country	Methodology	Population	Key findings
Martínez-Ruiz and Martínez-Mollá [17]	Embrace Cradles in the Process of Perinatal Grief/Las Cunas de Abrazos en la Elaboración del Duelo Perinatal	Spain	Systematic review of 8 articles (4 included in final analysis)	Spanish hospitals	Embrace cradles are viewed positively; they preserve the infant’s body, provide time for mourning, support bonding, and foster family unity
Ezzahra Azza Mazighi [7]	Nursing approach to perinatal death/Abordaje de enfermería en el duelo Ante una Muerte Perinatal	Spain	Bibliographic review (Final Degree Project, Universitat Rovira i Virgili)	16 articles from PubMed, CINAHL, Scopus, Cuidatge, Dialnet Plus, Cochrane, Cuiden, Teseo, and Google Scholar	No definitive procedure exists for perinatal death care; controversy remains over postpartum room location during hospitalization
Sánchez Villalba et al. [25]	Comprehensive support for parents in perinatal grief and emotional and psychological support tools for healthcare personnel/apoyo integral a los Padres en el Duelo Perinatal y Herramientas de Soporte Emocional y Psicológico en el Personal Sanitario	Spain	Literature review of articles from Medline, ScienceDirect, SciELO, PubMed, Dialnet, Cochrane, and WHO	Spanish hospitals	Advocates for a private farewell space marked with a blue butterfly symbol, free from staff interruptions, to create a calm, unhurried atmosphere with full family support
Hvidtjørn et al. [11]	Women’s length of stay in a Danish specialized unit for perinatally bereaved parents	Denmark	Population-based descriptive study	Women admitted between Jan 2012–Dec 2018	Unlimited stays in specialized units varied; primiparous women and those losing infants closer to term more often stayed up to 8 days, suggesting individual support needs beyond standard care
Roberts et al. [23]	Bereavement care guidelines used in health care facilities immediately following perinatal loss: a scoping review	USA (California)	JBI scoping review methodology	Sources on bereavement care guidelines post-miscarriage, stillbirth, or neonatal death, and parental mental health outcomes	Guidelines clustered into five categories: making meaning/memories, good communication, shared decision-making, emotional/social support, and organizational response (e.g., privacy, separate room, time with baby)
Camacho-Ávila et al. [4]	Experience of parents who have suffered a perinatal death in two Spanish hospitals: a qualitative study	Spain	Qualitative study using Gadamer’s hermeneutic phenomenology	13 mothers and 8 fathers bereaved within the past 5 years	Sharing hospital rooms with parents of healthy newborns intensified suffering; hearing babies and hospital routines was frustrating and distressing
Steen [26]	Raising the bar: development of a perinatal bereavement program	USA (Minnesota)	Descriptive intervention study	Families receiving specialized perinatal bereavement care	Program emphasized individualized care, memory-making, and allowing parents as much time as desired with their deceased baby, including opportunities for family and friends to meet the baby
Twigger-Ross and Uzzell [27]	Place and identity processes	UK (London)	Prospective cross-sectional descriptive multicenter study	20 residents interviewed	Place attachment linked to identity through continuity, self-esteem, self-efficacy, and distinctiveness; maintaining ties to places supports identity continuity
Pasca Garcia [21]	The conception of housing and its objects/La concepción de la vivienda y sus objetos	Spain	Master’s thesis	50 single-person households in Madrid	Strong relationship between identity and home attachment; control and security are key functions of place attachment
Kristensen et al. [15]	Different trajectories of prolonged grief in bereaved family members after terror	Norway	Longitudinal study at 18, 28, and 40 months post-event	129 parents and siblings bereaved in 2011 Utøya Island terror attack	Long-lasting grief included trauma symptoms and PGD; both early and long-term interventions are needed
Jørgensen et al. [14]	Stillbirth—transitions and rituals when birth brings death: data from a Danish national cohort seen through an anthropological lens	Denmark	Web-based questionnaires	173 bereaved parents (2015–2019)	Danish parents frequently spend extended time with their stillborn child, engaging in high levels of contact and memory-making

Adapted from multiples articles*

### Stage 5: collating, summarizing, and reporting the results

Two independent reviewers (RC and AO) screened all retrieved articles according to the inclusion and exclusion criteria. Disagreements were resolved through discussion or, if necessary, adjudication by a third reviewer (MG). Full-text articles of potentially relevant studies, as well as web-based materials and abstracts, were retrieved and independently assessed by the same two reviewers.

**Fig. 2:**
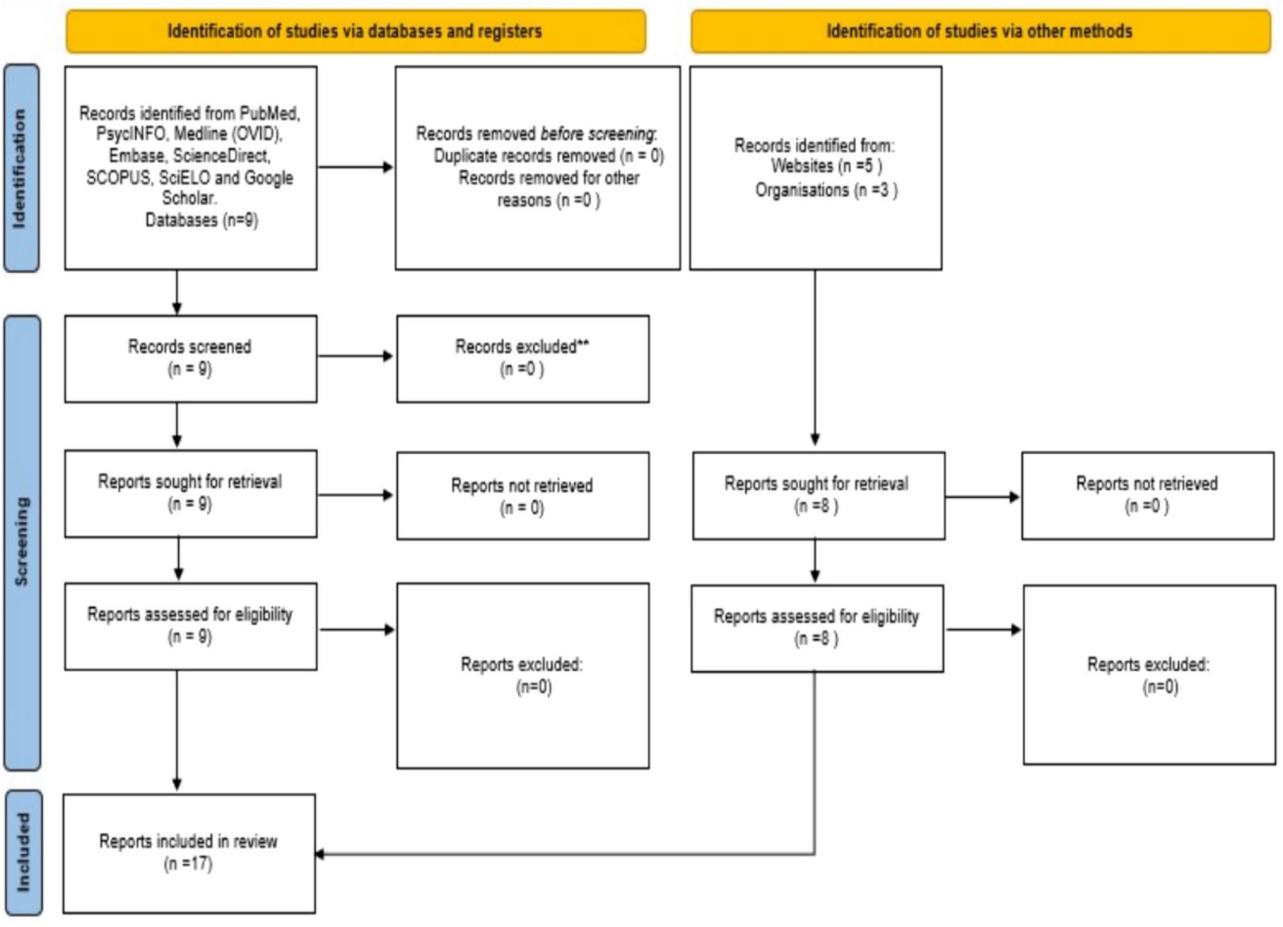
Flow diagram for scientific studies about perinatal loss and bereavement rooms

A PRISMA flow diagram (Fig. 2) was used to document the study selection process, the number of articles excluded at each stage, and reasons for exclusion.

**Fig. 3:**
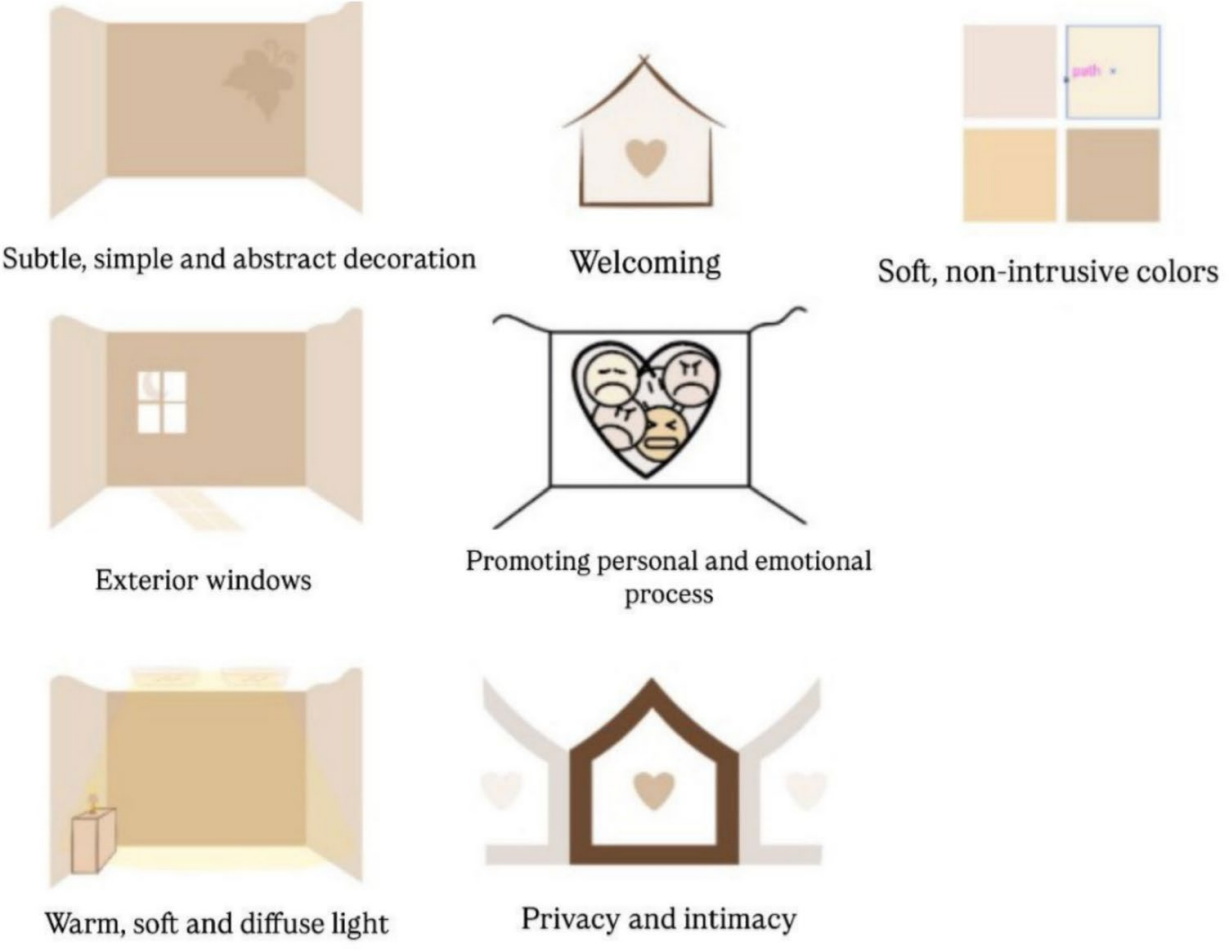
Bereavement room architecture

To ensure a multidisciplinary perspective, a review panel was convened comprising a psychiatrist (RC), three physicians (AO, MG, and LG), a psychologist (GS), a communications specialist (SE), and an architect with expertise in mental health design (AI). The architect’s contribution was particularly valuable in implementing a focused review methodology, examining architectural plans and studies with special attention to privacy considerations in the context of grieving rooms (Fig. 3). A detailed architectural analysis is provided in the section “Architectural perspective”.

## Results

While the term “*perinatal bereavement room”* appears in numerous articles and sources, few provide detailed descriptions of these spaces. Most publications focus on developing related programs and emphasizing the broader importance of managing perinatal grief; however, the specific implementation of bereavement rooms and their impact on families remain insufficiently addressed.

Of the 17 articles included in this review, only 4 (24%) explicitly discuss the potential benefits or perceived necessity of designated spaces for supporting perinatal grief. Even in these cases, the discussion is limited to general endorsements or brief mentions, without substantive detail on room layout, available resources, or the therapeutic environment.

Most studies merely acknowledge the existence or intended purpose of bereavement rooms, offering no evaluation of their implementation or effectiveness. Only one article (6%) provides a personal account from a postpartum woman who was required to share a room with mothers of healthy newborns following her loss; however, it does not explore supportive alternatives. Three articles (18%) note the need for clear protocols or supportive approaches related to bereavement spaces, yet none present concrete examples, best-practice guidelines, or evaluations of real-world implementation.

Several articles deviate from the central topic—for example, one focuses on prolonged grief disorder after traumatic loss without addressing perinatal grief spaces. Others contain only anecdotal references to bereavement rooms within healthcare settings, with no systematic research or standardized design guidance identified. These articles were retained for transparency, because they contained peripheral information relevant to the conceptual framework for perinatal grief care, even if they did not meet all topical criteria.

Overall, the literature demonstrates a clear lack of empirical data and practical recommendations for the creation, implementation, and evaluation of perinatal bereavement rooms, underscoring the need for targeted research to guide evidence-based design and policy development (Tables 3 and 4). For example, even the most detailed source identified—Roberts et al. [23]—provides only a brief architectural description without data on family mental health outcomes.

**Table 3:** Summary of articles on testimonies and experiences with perinatal/bereavement rooms

First author/reference	Title (English/Spanish)	Country	Article type	Population	Key findings
Calidad [3]	Hospital Federico Lleras Acosta opens the first perinatal grief room in the public network in the country/Hospital Federico Lleras Acosta abre la primera habitación de duelo perinatal de la red pública en el país	Colombia	News article	Mothers and families experiencing gestational or neonatal loss	The hospital inaugurated “Huellas de Amor,” a private, dignified space where grieving families can say goodbye, access memory boxes, and receive trained emotional support from an interdisciplinary care team
Alcantara [1]	Inaugurate first perinatal grief room in the country/Inauguran primera habitación de duelo perinatal en el país	Dominican Republic	News article	Parents and families losing a baby before or shortly after birth	The ADPP launched “Jardín Colibrí,” a comforting farewell room at Hospital Materno Infantil San Lorenzo de los Mina, enabling families to hold, dress, and say goodbye to their baby, alongside training for health professionals
Gallizo [9]	The home is one of the psychological anchors of the human being/La casa es uno de los anclajes psicológicos del ser humano	Spain	Opinion / Reflection	General population (residents, individuals attached to home)	The article explores the deep psychological significance of the home as a symbol of security, identity, and emotional anchoring for humans

**Table 4:** Independent associations establishing grief rooms for perinatal bereavement

Organization	Organization type	Country	Website
No foot too small	Non-profit/charitable organization	USA (Iowa)	https://www.nofoottoosmall.org/
The Hayden & Crue project	Non-profit initiative (family-founded)	USA (Ohio)	https://thehaydenandcrueproject.com
Jane’s room	501(c)(3) Non-profit organization	USA (Illinois)	https://janesroom.org/

## Discussion

Several articles identified in the review deviated from the central focus on perinatal bereavement rooms. For example, one examined prolonged grief disorder following traumatic loss without addressing perinatal grief spaces, while others referenced such rooms only briefly or without relevant detail. This scope divergence underscores the scarcity of targeted, high-quality research specifically examining the design, implementation, and impact of dedicated spaces for perinatal grief.

Perinatal loss—including miscarriage, stillbirth, and neonatal death—is widely recognized as a uniquely complex and devastating form of grief [8]. Such loss can shatter expectations, disrupt envisioned futures, and create profound emotional disorientation. It is often accompanied by isolation and stigma [31]. While grief-support programs in healthcare settings have been shown to reduce adverse mental health outcomes among parents, families, and providers, there is currently no direct evidence demonstrating the specific impact of bereavement rooms on psychological or emotional well-being.

Across all reviewed sources, no studies reported data on depression, anxiety, or posttraumatic stress disorder (PTSD) among families who have used bereavement rooms. None examined whether the presence or absence of such spaces has any measurable effect on the mental health or well-being of individuals experiencing perinatal loss. This absence of evidence is striking given the documented vulnerability of this population. Consequently, we were unable to address our primary review questions:

1. What is currently known about the role of physical spaces in the perinatal loss experience?
2. How does the availability of such spaces influence grief among individuals and families who have experienced miscarriage, stillbirth, or neonatal death?

The findings reveal a persistent gap in both the literature and clinical practice: the lack of detailed, empirical investigation into the physical, emotional, and practical roles of bereavement rooms. Although there is broad agreement—across guidelines [5], John [13] and anecdotal reports—that such spaces are valuable, current literature provides no standardized, evidence-based recommendations for their design, implementation, or evaluation. Importantly, no studies to date have established whether bereavement rooms improve measurable health outcomes for bereaved parents.

The statement that the “global availability of bereavement rooms remains limited” is based on preliminary, unpublished work by the WARMI Mental Health team, which has so far identified 32 such rooms in Latin America. We present this as observational context rather than as a definitive global statistic.

Future research should include both qualitative and quantitative approaches, addressing architectural and supportive features alongside clinical effectiveness in fostering psychological recovery and overall well-being. Research should also explore cultural differences in the use and meaning of such spaces, ensuring recommendations are relevant across diverse healthcare systems.

While bereavement rooms are not yet universally implemented and evidence for their measurable clinical benefits remains absent, the need for compassionate, high-quality care for families experiencing perinatal loss is unequivocal. Our recommendations in Table 5 are derived from a combination of existing guidelines and the interdisciplinary team’s professional experience in psychiatry, psychology, obstetrics, and architecture. Where based solely on expert consensus, this is explicitly stated.

**Table 5:** Concepts integrating architecture, psychology, psychiatry, and gynecology/obstetrics on bereaved mothers and families

The psychological needs of bereaved mothers and families				
Needs	Grief and trauma	Need for privacy and dignity	Emotional and social support	Opportunities for remembrance and meaning-making
Rational	Perinatal loss can result in intense grief, sadness, anxiety, and even posttraumatic stress disorder (PTSD)	Bereaved mothers need spaces where they can grieve privately and with dignity	Access to counseling, support groups, and spaces for connection with loved ones are vital	Spaces that facilitate remembrance of the lost child and help mothers find meaning can be beneficial
How architecture can support psychological needs				
Concept (s)	Creating a sense of safety and security	Designing comfortable and nurturing spaces	Connecting with nature and promoting mindfulness	Adapt logistical procedures
Practical expression(s)	Calming colors and soft lighting: using soothing colors and gentle lighting can create a tranquil and comforting atmosphere	Soft textures and comfortable furniture: incorporating soft textures and comfortable seating can create a sense of physical and emotional comfort	Indoor plants and natural light: bringing elements of nature indoors can have a calming and restorative effect	Improve the process for transferring deceased infants to the mortuary, using more appropriate and sensitive methods to minimize emotional impact
	Privacy and Noise Reduction: providing private spaces and minimizing noise can reduce anxiety and promote a sense of security	Personal Touches and Memory-Making Opportunities: including personal items, memory boxes, or spaces for reflection can help mothers grieve and remember	Designated Spaces for Mindfulness and Meditation: creating a quiet corner or space for reflection can encourage self-care and emotional regulation	Adapting procedures for the respectful transfer of deceased infants ensure more compassionate, family-centered care
Examples of integrating architecture and psychology				
	Hospital bereavement suites: these spaces can offer private rooms, comfortable furnishings, and areas for family gatherings	Healing gardens Outdoor spaces that incorporate nature can provide a calming and restorative environment	Support centers: these spaces can offer private counseling rooms, group meeting spaces, and areas for connection and community	Memorial spaces: designated areas or monuments can help mothers commemorate their lost child and find solace

Turning this recognition into reality will require coordinated action among policymakers, advocacy groups, healthcare professionals, and communities. Bereavement rooms should evolve from concept to *standard of care*—designed with evidence, implemented with consistency, and offered equitably—so that every family facing perinatal loss has access to an environment that supports dignity, connection, and healing.

## Architectural perspective

### The room as a protective environment in perinatal bereavement

Our review identified a significant gap in empirical research on bereavement rooms: of the 17 articles meeting inclusion criteria, only 4 (24%) referenced such spaces, and just 3 (18%) noted the need for formal protocols—without providing concrete examples. No studies evaluated implementation, design standards, or measurable impacts on grief, mental health, or family well-being. This lack of standardized guidance limits the ability to integrate therapeutic environments into perinatal bereavement care.

Against this backdrop, optimal care for families experiencing perinatal loss should begin immediately, supporting a grief process that promotes more favorable outcomes. “Generally, we think of rooms as places where one is sheltered and simply feels safe” [9], see Table 2). In this context, dedicated spaces can function as protective environments—providing safety, privacy, and a buffer from distressing external stimuli [20].

The concept of a “protective environment” is supported by literature on therapeutic spaces in healthcare [18]. Well-designed spaces—such as memorials and healing environments—can foster emotional processing and aid grief recovery. Incorporating natural elements, reflective surfaces, multisensory engagement, and opportunities for ritual has been shown to reduce stress and promote reflection by creating therapeutic atmospheres [28, 29]. These principles are further reinforced by the professional observations of our interdisciplinary team. Where our discussion extends beyond published findings—for example, in proposing optimal environmental configurations—recommendations are based on expert consensus rather than direct empirical study.

### Core characteristics of a perinatal bereavement room

Core characteristics of perinatal bereavement rooms include:

1. Privacy—separation from other patients or newborns while enabling necessary medical care within the room.
2. Comforting environment—warm decor, soft lighting, and comfortable furnishings that create a distinct atmosphere from standard hospital rooms.
3. Emotional support—on-site access to professionals trained in perinatal grief, such as psychologists, social workers, or psychiatrists.
4. Opportunities for rituals**—**space and resources for symbolic acts, such as photography, message writing, and preserving mementos.

These elements are grounded in existing guidelines [5], John [13], supported by literature on therapeutic spaces in healthcare [18], and consistent with qualitative findings that highlight the importance of professional presence for both parents and healthcare providers [12].

### Integrating architectural and psychological needs

The greatest impact arises when architectural design and psychological care principles are planned together from the outset. Bereavement rooms designed in alignment with compassionate care protocols can foster emotional safety, promote memory-making, and respect cultural or personal grieving practices.

Integration should consider:

- Soundproofing to shield families from distressing external noises.
- Adequate space with warm, adjustable lighting, comfortable temperature, and access to natural light.
- Neutral color palettes to encourage a calming atmosphere.
- Functional furnishings—including a hospital bed, seating, a bassinet, and a small table—that accommodate both medical needs and personal rituals.

Table 5 presents an original synthesis by the authors, derived from expertise in architecture, psychology, psychiatry, and obstetric care, and consistent with themes identified in the literature review.

By uniting design features with psychological support strategies, healthcare facilities can create environments that do more than house grief—they actively support healing, dignity, and emotional recovery.

## Conclusion

Perinatal bereavement rooms represent a compassionate and urgently needed response to the profound emotional and psychological impact of pregnancy and neonatal loss. Within perinatal grief programs, their absence represents a critical gap in both clinical practice and healthcare design. Although conceptually aligned with best practices in therapeutic healthcare environments, current literature offers minimal empirical guidance for their design, implementation, and evaluation. Of the 17 studies meeting our inclusion criteria, only 4 (24%) explicitly referenced bereavement rooms, and just 3 (18%) mentioned the need for formal protocols—none providing concrete examples. No studies evaluated the measurable impact of these spaces on grief recovery, mental health, or family well-being.

Despite this lack of quantified outcomes, consistent themes across guidelines, professional observations, and anecdotal accounts point to the profound potential of these environments to transform bereavement care. Purposefully designed bereavement rooms—integrating principles from architecture, psychology, and obstetric care—can do more than provide privacy. They can offer dignity, compassion, and a sense of control during moments when families often feel powerless. Such spaces create opportunities for connection, ritual, and memory-making, while reducing exposure to distressing stimuli and supporting the complex psychological needs that accompany perinatal loss.

Features such as privacy, soundproofing, access to natural light, neutral color palettes, and spaces for ritual—elements well documented in broader therapeutic healthcare literature—were seldom detailed in the perinatal bereavement context. Without formalized guidelines and implementation frameworks, these benefits remain inconsistently realized. The absence of robust evidence should not delay progress; rather, it should motivate targeted, multidisciplinary research to establish best-practice design guidelines and evaluate their effects on emotional recovery, family well-being, and staff support. Collaborative partnerships between healthcare providers, policymakers, researchers, and design experts can make bereavement rooms a standard, equitable feature of maternity and neonatal care, aligning with multidisciplinary perspectives on perinatal loss care [12].

By committing to environments that are both compassionate and evidence-informed, healthcare systems can ensure that no family faces perinatal loss without the shelter, safety, and dignity they deserve. In the face of unimaginable loss, we have the power—and the responsibility—to ensure that the space in which families grieve becomes part of their healing, not their harm.

## Data Availability

No datasets were generated or analyzed during the current study.
